# Activation of skeletal muscle mechanoreceptors and nociceptors reduces the exercise performance of the contralateral homologous muscles

**DOI:** 10.1152/ajpregu.00069.2024

**Published:** 2024-08-05

**Authors:** Fabio Zambolin, Fabio Giuseppe Laginestra, Thomas Favaretto, Gaia Giuriato, Matteo Maria Ottaviani, Federico Schena, Pablo Duro-Ocana, Jamie Stewart McPhee, Massimo Venturelli

**Affiliations:** ^1^Department of Sport and Exercise Sciences, Manchester Metropolitan University, Manchester, United Kingdom; ^2^Manchester Metropolitan University Institute of Sport, Manchester Metropolitan University, Manchester, United Kingdom; ^3^Department of Neurosciences, Biomedicine and Movement Sciences, University of Verona, Verona, Italy; ^4^Department of Internal Medicine, University of Utah, Salt Lake City, Utah, United States; ^5^Department of Neurosurgery, University Politecnica delle Marche, Ancona, Italy; ^6^Department of Life Sciences, Manchester Metropolitan University, Manchester, United Kingdom; ^7^Department of Anesthesia, Manchester University NHS Foundation Trust, Manchester, United Kingdom

**Keywords:** exercise-induced muscle damage, exercise performance, muscle nerve afferents, neuromuscular fatigue, stretching

## Abstract

Increasing evidence suggests that activation of muscle nerve afferents may inhibit central motor drive, affecting contractile performance of remote exercising muscles. Although these effects are well documented for metaboreceptors, very little is known about the activation of mechano- and mechanonociceptive afferents on performance fatigability. Therefore, the purpose of the present study was to examine the influence of mechanoreceptors and nociceptors on performance fatigability. Eight healthy young males undertook four randomized experimental sessions on separate occasions in which the experimental knee extensors were the following: *1*) resting (CTRL), *2*) passively stretched (ST), *3*) resting with delayed onset muscle soreness (DOMS), or *4*) passively stretched with DOMS (DOMS+ST), whereas the contralateral leg performed an isometric time to task failure (TTF). Changes in maximal voluntary contraction (ΔMVC), potentiated twitch force (ΔQ_tw,pot_), and voluntary muscle activation (ΔVA) were also assessed. TTF was reduced in DOMS+ST (−43%) and ST (−29%) compared with CTRL. DOMS+ST also showed a greater reduction of VA (−25% vs. −8%, respectively) and MVC compared with CTRL (−28% vs. −45%, respectively). Rate of perceived exertion (RPE) was significantly increased at the initial stages (20–40–60%) of the TTF in DOMS+ST compared with all conditions. These findings indicate that activation of mechanosensitive and mechanonociceptive afferents of a muscle with DOMS reduces TTF of the contralateral homologous exercising limb, in part, by reducing VA, thereby accelerating mechanisms of central fatigue.

**NEW & NOTEWORTHY** We found that activation of mechanosensitive and nociceptive nerve afferents of a rested muscle group experiencing delayed onset muscle soreness was associated with reduced exercise performance of the homologous exercising muscles of the contralateral limb. This occurred with lower muscle voluntary activation of the exercising muscle at the point of task failure.

## Graphical abstract

**Figure:**
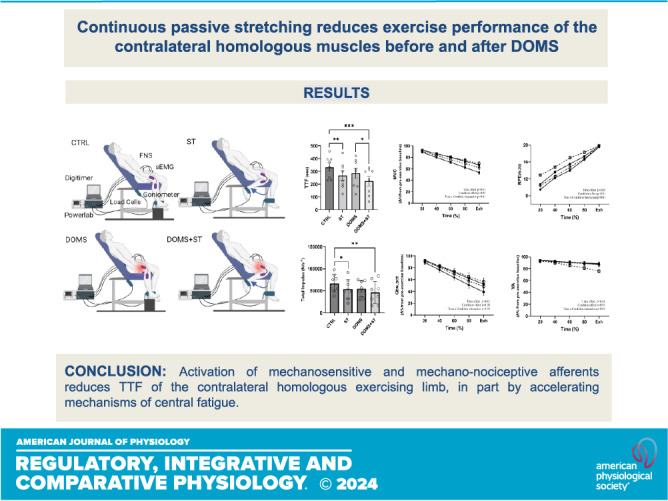

## INTRODUCTION

Performance fatigability has been defined as the acute decline in exercise performance over time (1, 2), including any reduction in the ability of muscles involved in the exercise to generate force or power. Performance fatigability can be classified into two main mechanisms, known as central and peripheral fatigue. Central fatigue affects the ability to generate and transmit motor commands, manifested as reduced motivation to continue the exercising task and activity in the motor cortex, as well as impaired descending drive to motor neurons (3). Peripheral fatigue includes all impairments occurring at or distal to the neuromuscular junction, affecting action potential propagation along muscle fibers and sarcomere cross-bridge function (4). In addition to the descending motor drive and muscle contractions, performance fatigability may be influenced by muscle sensory afferents that return information about muscle contractile activity back to the central nervous system. To date, the role of muscle afferents in performance fatigability remains unclear.

Group III–IV muscle nerve afferents (5) increase their discharge activity in response to mechanical (mechanoreceptors), metabolic (metaboreceptors), and nociceptive (nociceptors) stimuli within the muscle microenvironment (6–8). Their activity increases pulmonary ventilation and muscle blood flow to sustain exercise or adjust to new workloads. Past studies have demonstrated how III–IV muscle afferents activation stimulates respiratory neurons to increase breathing rate and depth (9), and autonomic nervous system activation to increase heart rate and cardiac output (10). Conversely, activity of III–IV muscle nerve afferents can also restrict the outputs of spinal motoneurons, reducing motor unit recruitment and contributing to the loss of force (5, 11). However, there is conflicting evidence about the contribution of muscle nerve afferents to the development of central fatigue. Some studies reported no change in corticospinal outputs when III–IV afferents were stimulated (12, 13), whereas others reported reduced voluntary activation (14–17) with further differences in corticospinal motor evoked potential between elbow flexors and extensor muscles (18). The conflicting findings may have been due to differences in the exercise context, such as intensity, whether the whole body or only a single limb was active, whether blood flow was maintained throughout exercise or if it was occluded, and the prior exercise history that may involve the presence of muscle damage. To understand the effects of muscle afferents during exercise and whether it is a factor to performance fatigability, it is important to discern the effects of each specific afferents (i.e., mechano-, metabo-, nociceptive) under consideration.

Distinguishing between III and IV afferents involves some methodological challenges in exercising humans. Past studies inflated a cuff to occlude blood flow or injected hypertonic saline solutions to stimulate muscle metaboreceptors and nociceptors without further activating mechanoreceptors (13, 15, 17). However, these approaches do not demonstrate effects of metabo- or nociceptors activity in isolation because metabolite accumulations can sensitize mechanoreceptors causing them to increase their activity to any given mechanical stimulus (19, 20). An alternative approach used to determine the contribution of mechanoreceptors without further activating metaboreceptors consisted of applying a passive static stretch to a rested muscle (21). Passive stretching provides a mechanical stimulus without incurring metabolite accumulations (22) and could be used to examine the role of mechanoreceptors on performance fatigability and their mechanisms of action. However, it is not possible to passively stretch a muscle that is voluntarily developing force during exercise, so the stretch must be applied to another muscle not involved directly in the exercise. To this end, we recently showed that a passive stretch applied to the rested knee extensors of one leg leads to changes in the peripheral and central hemodynamics, even when measured in the contralateral leg (23). We further showed that effects were greater when the stretch was applied to a muscle affected by delayed onset muscle soreness (DOMS) (23), which sensitizes mechanoreceptors and nociceptors (24, 25) without necessarily altering the metaboreceptors activity (26, 27). These findings suggest that activating mechanoreceptors of resting muscles may affect the function of another active muscle, and that the presence of DOMS may exacerbate these effects (28).

Therefore, the purpose of the present study was to examine the influence of mechanoreceptors and nociceptors on performance fatigability. A randomized, controlled, crossover trial using a unilateral knee-extension paradigm was conducted. It was hypothesized that the passive static stretch applied unilaterally to the rested knee extensors would reduce the performance of the contralateral leg during a fatiguing exercise task. The passive stretch was expected to reduce voluntary activation at the point of task failure. Effects were expected to be elevated when the static stretch was applied to a muscle experiencing DOMS when the mechanoreceptors and nociceptors were sensitized.

## METHODS

### Participant Characteristics

Eight male volunteers (age: 24 ± 2 yr; body mass: 72 ± 10 kg; and height: 179 ± 8 cm) took part in the study. Participants were healthy, nonsmokers, and recreationally active (3.0 ± 0.5 h of exercise per week) but with no specific experience of strength training exercise. The study received ethical approval from the ethical committee of the University of Verona (CARP: Ref. No. 14 R2/2021; No. 37464) and conformed to the Declaration of Helsinki. The participants gave written, informed consent before their participation after full explanation of the purpose and experimental procedures of the study. The participants reported to the laboratory in the morning (8:00–9:00 AM) in a fasted state. They were instructed to refrain from taking caffeine for at least 24 h before the testing session and to report to the laboratory without engaging in any sort of strenuous physical activity in the preceding 48 h. Participants were also restricted from eating any vitamin supplements, high-vitamin C foods, alcohol, or pain medicines within 24 h of each visit.

### Experimental Design

The experimental design of this study consisted of a total of seven different laboratory visits. Two familiarization visits were performed to ensure that participants were familiar with the time to task failure (TTF). Thereafter, the participants reported to the laboratory for five different laboratory visits, in which they performed one of the four experimental sessions on different days [Control (CTRL); Stretching (ST); DOMS; DOMS with stretching (DOMS+ST)] and the exercise-induced muscle damage (EIMD) protocol. The EIMD protocol was always performed 24 h before the participant was randomized to complete the DOMS or DOMS+ST condition. The CTRL condition consisted of a TTF (described in further detail later) performed on the dominant leg, whereas the contralateral leg was resting flexed and fully relaxed over a wooden box. ST consisted of the same TTF on the dominant leg, but performing the static stretching protocol (described in further detail later) on the contralateral leg. DOMS condition consisted of the same TTF test on the dominant leg, whereas the contralateral leg was resting fully relaxed after having previously performed the EIMD protocol. DOMS+ST conditions consisted of the same TTF on the dominant leg, whereas in the contralateral leg a static stretching protocol was applied after the previous EIMD protocol. The order of each condition (CTRL vs. ST and DOMS vs. DOMS+ST) was randomized and counterbalanced between participants. Each session was performed on a separate day with DOMS and DOMS+ST conditions performed randomly and counterbalanced at 24 and 48 h after the EIMD protocol. CTRL and ST conditions were similarly spaced to DOMS and DOMS+ST protocol (i.e., 24 h). When the EIMD protocol was performed first, CTRL and ST were performed following a period of washout (7–10 days) when participants were fully recovered from EIMD.

### Femoral Nerve Stimulation

The motor nerve was stimulated via a constant current electrical stimulator (Digitimer DS7AH, Welwyn Garden City, UK) with the anode placed between the greater trochanter and the iliac crest and the cathode placed over the femoral nerve in the femoral triangle. Electrical stimuli were delivered through circular (32 mm) self-adhesive electrodes (Facial Electrodes, Globus G0451). The evoked twitch force was measured by a force transducer (model UU2; DaCell, Korea) previously calibrated and connected to a custom-made chair through a noncompliant strap placed around the ankle (29). The ankle was firmly connected to the load cell to provide full force transfer at the delivery of the twitch. The load cell was calibrated before each experiment and the baseline was set up accounting for the initial tension of the leg for each participant. The participants were seated with a 90° knee flexion with the hip extended at 135–140° and ensuring a posterior pelvic tilt between 80 and 90°, securely strapped to avoid postural changes during the time to task failure and across conditions. The output from the load cell was amplified (INT2-L; London Electronics Limited, Sandy Bedfordshire, UK) and recorded at a sampling rate of 2 kHz. Once the electrodes were in place, stimulation intensity was increased by 25 mA increments until the size of the single evoked twitch force demonstrated no further increase despite an increase in the stimulating current. Stimulation current was then set at 125% of the supramaximal value (means ± SD: 360 ± 10 mA for CTRL, 365 ± 8 mA for ST, 358 ± 11 mA for DOMS, and 369 ± 9 mA for DOMS+ST). The interpolated twitch technique was used to evaluate knee extensor voluntary activation (VA): a supramaximal twitch was superimposed at the moment of peak force during a maximal voluntary contraction (MVC) effort, and a second twitch was applied 2 s after relaxation from the MVC. This was repeated for MVCs tested at baseline and during the TTF. VA was estimated by expressing the additional force produced from the superimposed twitch (t) relative to that of the potentiated resting twitch (T) using the formula [100 × (1 – t/T)].

### Surface Electromyography

Vastus lateralis (VL) EMG was recorded on both the exercising and stretched limbs using a dual bioamplifier [ML135; ADInstruments, Bellavista, NSW, Australia; input impedance: 200 MΩ differential and 500 pF (supplied Bio Amp cable and leads) to isolated ground, common-mode rejection ratio >85 dB typical (1–60 Hz)]. On each VL, two surface Ag/AgCl electrodes (PG10C; FIAB, Vicchio, Italy) were attached to the skin with a 20-mm interelectrode distance. The electrodes were placed longitudinally on the VL muscle belly, in line with the underlying muscle fascicle arrangement, at two-thirds of the distance between the anterior iliac spine and the lateral part of the patella. The reference electrode was placed on the patellar tendon. Before the application of the electrodes, the skin was shaved, lightly abraded with sandpaper, and then cleansed with an alcohol swab to minimize impedance. The electrode location was marked on the skin with indelible ink to maintain placement constant across visits. The raw EMG signal was acquired at 2 kHz sampling frequency and stored for offline analysis. Acquisition of the EMG data was made using a computer-based data acquisition and analysis system [hardware: PowerLab 16/30 (ML880; ADInstruments) and software: LabChart8 (ADInstruments)].

### Time to Task Failure

**Figure 1.**
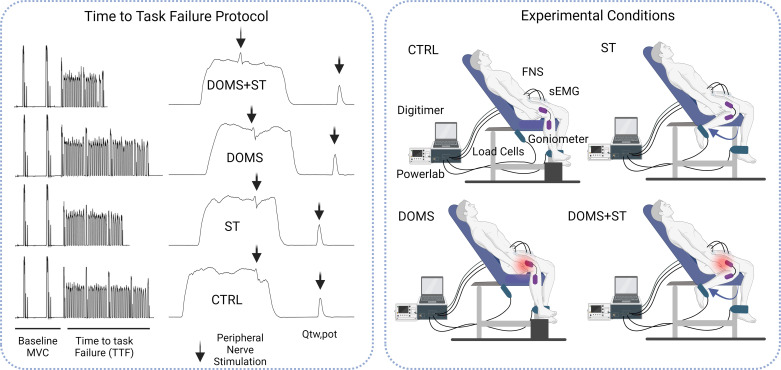
Experimental condition and time to task failure protocol*. Left*: illustrative data from each condition. *Right*: schematic of the experimental conditions. The TTF was performed with the dominant leg, whereas the nondominant leg either rested (CTRL and DOMS conditions) or was stretched (ST and DOMS+ST conditions). The TTF included repeated isometric knee-extension exercise at 45% MVC (60% duty cycle) to task failure. CTRL, control condition; DOMS+ST, delayed onset muscle soreness with stretching condition; DOMS, delayed onset muscle soreness condition; FNS, femoral nerve stimulation; MVC, maximal voluntary contraction; sEMG, surface electromyography; ST, stretching condition; TTF, time to task failure. Created with BioRender.com.

Participants were seated in a comfortable custom-made chair with the hip extended at 135–140°, whereas the knee angle of the dominant limb was fixed at 90°. After a warm-up consisting of four blocks of three repetitions with 10 s rest at increasing intensity (moderate to hard intensity), participants performed two MVCs separated by 2 min of recovery. After the warm-up and two baseline MVCs, participants then performed the full TTF protocol that consisted of blocks of 10 repeated isometric knee-extension contractions at 45% MVC with a 60% duty cycle (repeated bouts of three contraction and 2 s rest). Each set of 10 contractions was followed by an isometric MVC lasting 3 s, which also included superimposed and resting potentiated twitches to estimate the voluntary activation (as described earlier). Visual feedback was given to participants with boundaries of ±5% to match the 45% initial MVC target force during the time to task failure. Blocks of 10 contractions continued until participants reached task failure, which was defined as the moment when participants were not able to achieve the 45% MVC force for two consecutive attempts despite strong verbal encouragement (Fig. 1). Rate of perceived exertion (RPE) was measured every minute through the TTF with the Borg Scale (rating 6–20) (30) and used to confirm maximal effort from participants close to task failure (19–20). During the TTF in ST and DOMS+ST conditions, the contralateral leg was stretched at the same range of motion set during the familiarization visit (refer back to the static stretching protocol description), whereas in CTRL and DOMS conditions the leg was resting and supported using a wooden box (Fig. 1). Each TTF was determined independently in each of the four experimental sessions.

### Static Stretching Protocol

During the familiarization visit, participants’ maximum knee flexion range of movement (ROM) on the nondominant limb was tested in a posture comparable with the TTF experiment. All assessments were conducted by the same operator, who moved the participant’s nondominant joint through a 50° range of motion (knee flexion) until reaching the point of tolerable maximal flexion, where subjective tension was rated using a visual analog scale (VAS) and P-NRS scale (31). The range of tolerable maximal flexion was defined as the maximum tension needed to achieve minor self-perceived pain (i.e., <3 out of 10). Postural discomfort was assessed using a level of 3 (tolerable) on a 1–10 visual analog scale (32). VAS was used to self-report perception of stretching intensity using a 100-mm scale in which participants rated their perception of stretching intensity. Moreover, the subjective feeling of pain was recorded with the P-NRS scale, rating the pain arising from the stretching protocol from 0 (no pain at all) to 10 (pain as bad as it could be). Static stretching consisted of a single passive knee flexion lasting the whole trial. During the stretching protocol, knee joint angle was continuously recorded using a biaxial electrogoniometer (Twin axial goniometer TN1750/ST ADInstruments Systems, Oxford, UK) while the knee extensors were stretched to the same range of motion obtained during the initial ROM assessment (33), and this was kept identical for all stretching conditions (ST and DOMS+ST, respectively). An adjustable load cell was also fixed on the participant’s nondominant ankle and held during the experiment by a trained operator using the support of a rigid frame of the dynamometer to apply a consistent stretch and measure the force applied from the flexed, stretched nondominant leg during the entire protocol as previously described (23).

### Exercise-Induced Muscle Damage Protocol

A warm-up of 10 nondominant single-leg isokinetic knee extensions and knee flexions (corresponding to alternating concentric and eccentric muscle contractions) were carried out through the full test range of motion, ensuring a progressive increase in effort, using an isokinetic dynamometer (Cybex II, division of Lumex, Inc., Ronkonkoma, NY). A single-leg maximal isometric voluntary contraction of the nondominant leg was assessed prior to the EIMD protocol when participants were instructed to exert the maximal voluntary isometric contraction at 90°. The EIMD protocol consisted of several blocks of 3 sets of 12 maximal voluntary eccentric single knee extensions of the nondominant limb and 30 s of recovery followed by a postblock assessment of the maximal force. The exercise was stopped once all participants reached a reduction of >40% of the maximal force from baseline (27, 34). The eccentric phase of the contractions was performed at an angular velocity of 90°·s^−1^. The concentric phase was performed submaximally at an angular velocity of 90°·s^−1^ and resistance applied from the participants was negligible.

### Measures of Delayed Onset Muscle Soreness

To ensure the presence of DOMS after the EIMD protocol, a series of tests were carried out before the femoral nerve stimulation protocol for each condition (Fig. 1). Participants were fully rested before starting the trial. Muscle soreness was assessed by maximal voluntary contraction (MVC), using an isokinetic dynamometer (Cybex, division of Lumex, Inc., Ronkonkoma, NY) (35) and a 100-mm visual analog scale (VAS) anchored on the left edge of the scale with the phrase “no pain or soreness” and on the right edge “worst pain/soreness imaginable.” Participants were asked to rate their pain-related soreness after performing a body weight squat at ∼90° knee angle (VAS_SQ_) (35). Pain pressure thresholds (PPTs) were assessed using a marker placed on a standardized point of the nondominant quadriceps on the first day of testing to ensure reliable measurements between testing sessions (36). To determine PPTs, a standardized mechanical force gauge was used (Hilitand, NK-100 Force Gauge; measuring range: 10–100 N/1–10 kg; load division value: 0.5 N/0.1 kg). The same operator used the force gauge, applying a gradual force over a surface of 1 cm^2^ on the marked spot of the participant’s quadriceps until the participant verbally informed the researcher when the sensation became painful, and the PPTs were recorded (36). Force values, in kilograms, were converted to kilopascals and reported in Table 1.

**Table 1.** Measures of DOMS and neuromuscular function before the start of repeated isometric knee-extension exercise at 45% MVC (60% duty cycle) to task failure

Variable	CTRL	ST	DOMS	DOMS+ST
MVC nondominant limb (*N*)	685 ± 120	682 ± 130	420 ± 170**#	411 ± 197**#
PPTS, kPa	598 ± 127	578 ± 137	329 ± 117**#	382 ± 147**#
VAS squat, mm	0.4 ± 0.3	0.7 ± 0.2	46.5 ± 28.1**#	51.0 ± 20.4**#
Baseline MVC (*N*)	759 ± 129	678 ± 131	715 ± 126	682 ± 87
Baseline VA, %	94 ± 4	92 ± 4	93 ± 4	92 ± 5
Baseline Q_tw,pot_ (*N*)	295 ± 42	271 ± 50	289 ± 55	302 ± 65

Data are presented as means ± SD. CTRL, control condition; DOMS, delayed onset muscle soreness condition; DOMS+ST, delayed onset muscle soreness with stretching condition; MVC nondominant, maximum voluntary contraction of the nondominant limb performing the EIMD protocol; PPTS, pain pressure thresholds; Q_tw,pot_, quadriceps twitch potentiated; ST, stretching condition; VA, voluntary activation; VAS, visual analog scale. **P < 0.01 compared with CTRL; #*P* < 0.05 compared with ST.

### Data Analysis

Neuromuscular function was assessed as percentage changes from initiation of the TTF (MVC; VA; Q_tw,pot_). Total impulse was calculated as the force-time integral of the TTF. MVC values were calculated as the mean average force 500 ms before stimulation, applying a low-pass digital filter at cut-off frequency of 50 Hz, with auto-adjust function for transition width, using LabChart (v. 8.1.16; ADInstruments Systems, Oxford, UK) (13). LabChart Digital Filter is a zero-phase-lag finite impulse response (FIR) filter. FIR filters use a Kaiser window with β = 6, which results in pass and stop band ripple of <0.5%. The auto-adjust filter sharpness allows a cut-off width at 20% of the cut-off frequency. For each participant, the highest measured force during the baseline MVCs, and Q_tw,pot_ for each condition was calculated and used to normalize all the subsequent MVC and Q_tw,pot_ to this value (16, 29). MVC and Q_tw,pot_ were analyzed with LabChart Pro software (v. 8.1.16; ADInstruments Systems, Oxford, UK). EMG data were analyzed with a custom-built MATLAB routine (MATLAB 2020b; MathWorks, Natick, MA). Briefly, the raw EMG signal was band-pass filtered (10–450 Hz) with a fourth-order Butterworth filter and full-wave rectified. Afterward, a 250-ms baseline was detected between each knee extension, and contraction onset was defined as the point when the signal deviated by 3 standard deviations (SDs) from baseline. The same calculation was applied to find the contraction offset. For each muscle contraction, the average root mean square of the EMG signal (EMG_RMS_) for the VL muscle was calculated and normalized to the maximum EMG_RMS_ obtained during the baseline MVC. Maximum EMG_RMS_ was calculated from a 500-ms window preceding the superimposed electrical stimulation. Successively, EMG_RMS_ data from the knee-extension contractions to 45% MVC target force during the last 30 s of each minute of the TTF task were averaged together (37).

### Statistical Analysis

Sample size calculation was performed with G*Power (v.3.1.9.7) using the TTF as primary outcome based on data from Ref. 29, which compared TTF in conditions of preexisting fatigue versus control. Effect size was determined from the differences between pre- to posttask failure data, aiming for a power (1 − β err prob) of 0.95 and α level set at 0.05. Results from power calculation analysis using one-way repeated measures ANOVA for four groups were as follow (λ = 174.24; *F* = 2.79 and actual power = 1.0). This resulted in an estimated sample size of *n* = 8 participants. Normal distribution of the data was assessed with a Shapiro–Wilk test. Student’s paired *t* test was used to determine differences between ST and DOMS+ST conditions for VAS pain, VAS stretching intensity, and stretching measurements (ROM and force). A two-way repeated-measure ANOVA was performed to assess decline of neuromuscular and perceptual outcomes (MVC, VA, Q_tw,pot_, EMG, and RPE) during time (20%, 40%, 60%, 80%, exhaustion of the TTF) and between conditions (CTRL, ST, DOMS, DOMS+ST). All data were analyzed using the statistical software package GraphPad Prism v.10 (GraphPad Software, San Diego, CA) and are presented as means ± SD, unless otherwise stated, and were considered significant when *P* < 0.05.

## RESULTS

### Application of the Static Stretching

All participants completed the experimental procedures and laboratory sessions. Pain intensity rated with VAS at the start of the testing session was higher for DOMS+ST compared with ST (7.0 ± 2.6 vs. 2.6 ± 1.9 cm, respectively; *P* < 0.01). Participants reported very low (2/10 on a subjective numeric rating scale) or absent postural discomfort during the stretching procedures (32). Participants were set at the same flexed knee angle during ST and DOMS+ST conditions (54.9 ± 4.9° vs. 55.1 ± 4.7°, where full knee extension was 180°), and the force of the contralateral leg detected by the load cell when the rested knee was passively stretched was lower in ST compared with DOMS+ST conditions (67.1 ± 22.2 vs. 77.4 ± 24.5 N; *P* = 0.82). VAS for stretching intensity, which was rated during the application of the passive stretch, was similar for ST and DOMS+ST conditions (7.1 ± 1.3 vs. 8.1 ± 1.6 cm, respectively, *P* = 0.72). No muscle activity (EMG_RMS_) was detected during each condition.

### Measures of Delayed Onset Muscle Soreness

Data from measures of DOMS are reported in Table 1. MVC of the experimental limb (the leg that was stretched and/or had DOMS) differed significantly between conditions (*F* = 12.94, *P* < 0.01). It was similar for DOMS and DOMS+ST conditions (*P* = 0.45) but lower in DOMS (*P* < 0.01, *P* = 0.03) and DOMS+ST (*P* = 0.01, *P* = 0.04) compared with CTRL and ST conditions. PPTS differed significantly between conditions (*F* = 11.45, *P* < 0.01). It was lower in DOMS (*P* < 0.01, *P* = 0.02) and DOMS+ST (*P* < 0.01, *P* = 0.02) compared with CTRL and ST conditions but similar between DOMS and DOMS+ST conditions (*P* = 0.32). VAS_SQ_ differed significantly between conditions (*F* = 9.89, *P* < 0.01). It was higher in DOMS (both *P* = 0.02) and DOMS+ST (all *P* < 0.01) compared with CTRL and ST conditions, with no differences between DOMS and DOMS+ST (*P* = 0.49).

### Performance Fatigability during the TTF

**Figure 2.**
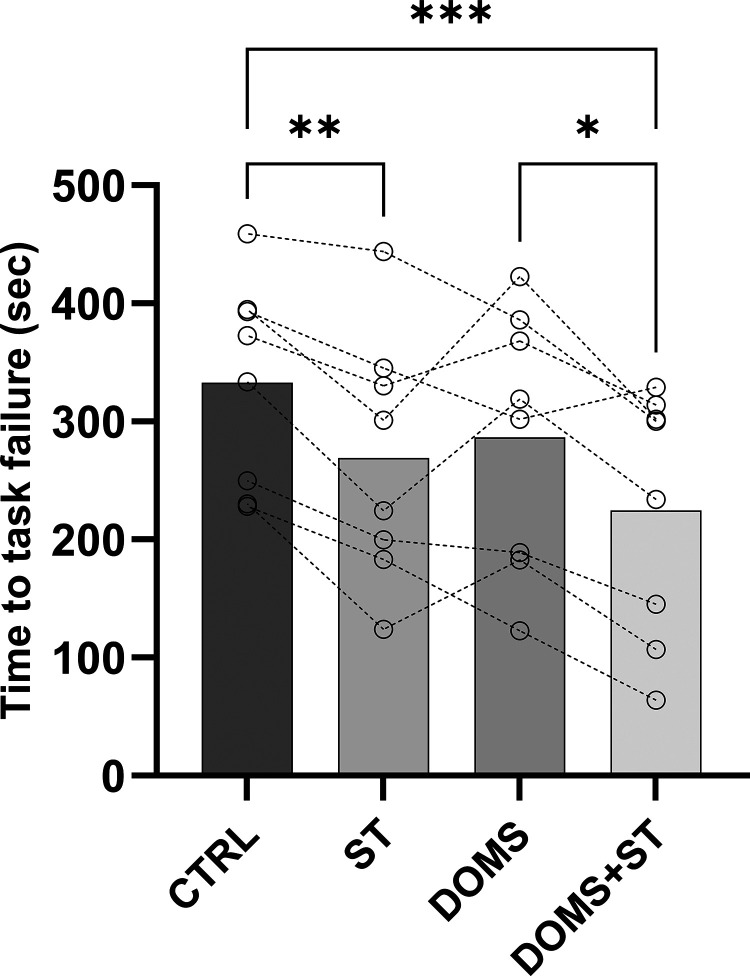
The time to task failure (TTF). CTRL, control condition; DOMS, delayed onset muscle soreness condition; DOMS+ST, delayed onset muscle soreness with stretching condition; ns, not significant; ST, stretching condition. *P < 0.05; **P < 0.01; ***P < 0.001. Data are presented as means ± SE, with individual data points shown as open circles.

Before starting the TTF with the contralateral knee extensors, there were no differences between conditions for MVC [*F*(1.800, 12.60) = 2.022, *P* = 0.17, Table 1], VA [*F*(2.354, 16.47) = 0.4998, *P* = 0.64, Table 1] or for Q_tw,pot_ [*F*(2.434, 17.04) = 1.883, *P* = 0.18, Table 1]. However, by the end of the fatiguing task, the TTF was significantly different between conditions [*F*(1.75, 12.27) = 12.83, *P* < 0.01]. It was lower in ST and DOMS+ST compared with CTRL (all *P* < 0.01; Fig. 2) and was lower in DOMS+ST compared with DOMS (*P* = 0.01). TTF did not differ significantly between DOMS and ST conditions (*P* = 0.21; Fig. 2). Total impulse was significantly different between conditions (*F* = 7.084, *P* < 0.01) and was lower in ST (50,850 ± 24,387 N·s, *P* = 0.02) and DOMS+ST conditions (49,817 ± 23,757 N·s, *P* = 0.01) compared with CTRL (66,931 ± 20,197 N·s). The DOMS condition (54,894 ± 17,967 N·s) did not differ from the other conditions.

### Neuromuscular Function during the TTF

MVC showed a significant effect of time [*F*(1.026, 28.72) = 461.4, *P* < 0.01], condition [*F*(3, 28) = 3.936, *P* = 0.02] and time × condition interaction [*F*(12, 112) = 3.966, *P* < 0.01]. Post hoc analysis showed lower reduction in MVC in DOMS+ST and ST conditions compared with CTRL at 40, 60, 80, and 100% of the TTF (all *P* < 0.05), whereas no differences were found on the post hoc comparison between any other time points or conditions. VA declined significantly over time [*F*(1.046, 29.29) = 73.84, *P* < 0.01], with no significant effect of condition [*F*(3, 28) = 1.235, *P* = 0.31]. However, VA showed a significant time × condition interaction [*F*(12, 112) = 9.118, *P* < 0.01]. Post hoc analysis showed a reduction in VA only in DOMS+ST compared with CTRL at 100% of the TTF (*P* = 0.02), whereas no differences were found on the post hoc comparison between any other time points or conditions. Q_tw,pot_ declined significantly over time [*F*(1.071, 29.98) = 237.6, *P* < 0.01] with no significant effect for condition [*F*(3, 28) = 1.061, *P* = 0.38] or time × condition interaction [*F*(12, 112) = 1.183, *P* = 0.30]. Q_tw,pot_ post hoc analysis showed no difference between time points or conditions. EMG_RMS_ showed a significant effect of time [*F*(2.107, 50.57) = 164.7, *P* < 0.01] with no significant effect for condition [*F*(3, 24) = 0.1740, *P* = 0.17] or time × condition interaction [*F*(12, 96) = 1.065, *P* = 0.39]. EMG_RMS_ post hoc analysis showed no difference between time points or conditions. RPE increased over time [*F*(3.063, 85.77) = 1,001, *P* < 0.01], showing a significant effect for condition [*F*(3, 28) = 57.18, *P* < 0.01] and time × condition interaction [*F*(12, 112) = 6.091, *P* < 0.01]. Post hoc analysis showed that RPE was significantly different in DOMS+ST compared with CTRL, ST, and DOMS at 20, 40, and 60% of the TTF (all *P* < 0.05). Significant changes in RPE were also present in ST compared with CTRL and DOMS condition at 40 and 60% of the TTF, whereas no differences were found on the post hoc comparison between any other time points or conditions.

## DISCUSSION

In this study, we used a model of unilateral knee-extension exercise in a randomized crossover design to provide novel insights into the possible role of muscle mechanoreceptors and nociceptors on performance fatigability. We found that a passive, static stretch applied to a rested muscle to activate mechanoreceptors caused a reduction in the TTF of the exercising knee extensors of the contralateral limb. When a stretch was applied to the rested muscles affected by DOMS to further activate mechanonociceptors, the reduced TTF was also accompanied by reduced VA and a smaller reduction of the Q_tw,pot_. In contrast, the presence of DOMS without stretch in the rested leg did not affect TTF of the contralateral exercising leg. These original findings support the hypothesis that activation of mechanoreceptors and mechanonociceptors contributes to performance fatigability. Furthermore, activation of muscle afferents during DOMS+ST seems to reduce voluntary activation during the TTF.

**Figure 3.**
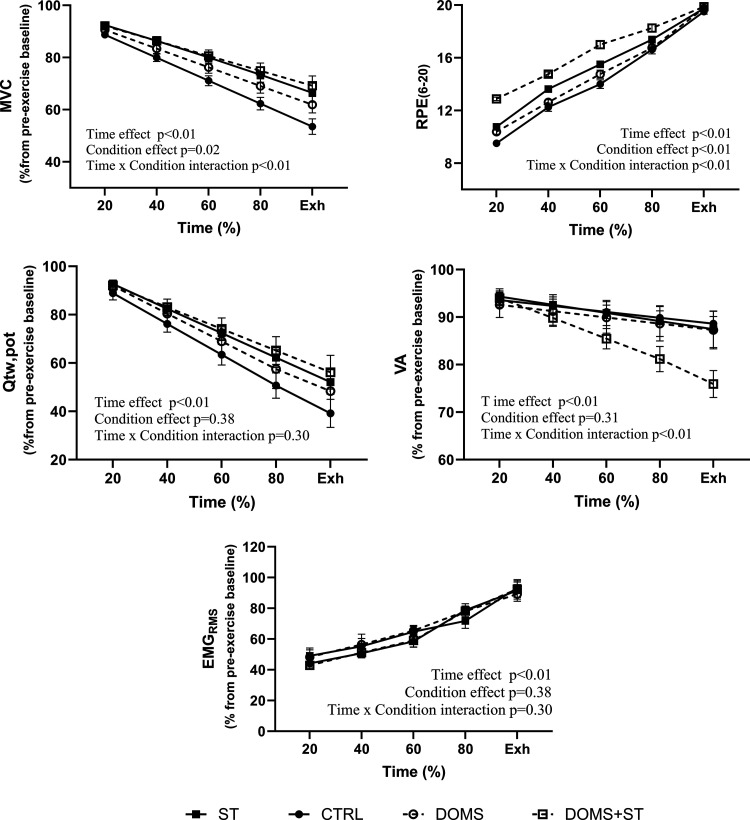
Delta and slopes changes of neuromuscular function following the repeated isometric knee-extension exercise at 45% MVC (60% duty cycle) to task failure. CTRL condition is represented with black circles and lines; ST condition is represented as black squared and lines; DOMS is represented as empty circles and dashed lines; DOMS+ST condition is represented with empty squared and dashed lines. CTRL, control condition; DOMS, delayed onset muscle soreness condition; DOMS+ST, delayed onset muscle soreness with stretching condition; Exh, exhaustion; MVC, maximal voluntary contraction; Q_tw,pot_, resting potentiated twitch; ST, stretching condition; TTF, time to task failure; VA, voluntary activation. Data are presented as means ± SE.

Performance fatigability develops due to impairments of the central motor drive and/or the peripheral muscle function. It is often characterized by a reduction in the MVC, and the results shown in Fig. 3 demonstrate progressive loss of MVC throughout exercise in all conditions. The methodology of the present study provides insights into the mechanisms of action when mechanosensitive afferents of the resting leg were activated by passive stretching. The resulting reduction of TTF in ST and DOMS+ST conditions was associated with a higher RPE throughout the majority of the exercise task, and a lower VA in DOMS+ST only, at task failure. These findings suggest that activation of mechanonociceptors of a resting muscle group affected by DOMS reduces TTF of the exercising homologous muscles of the contralateral limb through reduced voluntary activation. The fact that Q_tw,pot_ was not different from CTRL when stretch was applied, or better preserved in the case of DOMS+ST, suggests that activating the mechanonociceptors of the rested knee extensors did not accelerate aspects of peripheral fatigue.

Several past studies also examined the contributions of muscle afferents on performance fatigability. For instance, activation of mechano and metaboreceptors (i.e., group III and IV muscle nerve afferents) decreased the voluntary activation in knee extensor muscles during isometric tasks in the ipsilateral limb (17) and attenuated the decline in MVC force and Q_tw,pot_ in the contralateral homologous limb following previous ipsilateral electrical stimulation (29). Other recent investigations also showed contributions of metabo- and nociceptors activation to performance fatigability using blood flow cuff occlusion, hypertonic saline injection on the ipsilateral and contralateral limbs during time to task failure (13, 15, 17, 38). Some reported no reduction in voluntary activation and thus no direct contribution of central fatigue on the contralateral exercising limb when using blood flow cuff occlusion with whole body and isometric exercises (13, 17). Others did find decreases in voluntary activation following the injection of hypertonic saline into selected ipsilateral and contralateral muscles to activate nociceptors (15). These past studies report inconsistent findings, but our results are clearer, demonstrating inhibition of the central neural drive (i.e., lower voluntary activation) (4) when mechanonociceptors are activated, suggesting that nociceptors and pain might play an important role in VA reduction during time to task failure on ipsilateral but also contralateral limbs.

From our findings, it is not possible to determine the mechanisms leading to the decrease of VA after activation of mechanonociceptors. One possibility is that mechanoreceptor activity in stretched muscles affected by DOMS inhibits the contralateral homologous muscle spinal motor neurons, leading to a reduced motor neuron output for the active homologous muscle group (39). This might have caused a reduced spinal reflex excitability on the contralateral active muscle, showing a general inhibitory mechanism acting on motor neurons (40, 41). However, given that this effect was small in repeated dynamic passive stretching (39), further research needs to address this possibility by implementing continuous static stretching. Another possibility is that the elevated mechanonociceptor activity of stretched muscles affected by DOMS causes inhibition of supraspinal neurons controlling activation of the contralateral homologous active muscles to decrease the descending motor drive and accelerate the onset of a theoretical sensory tolerance limit. This occurs during maximal or prolonged exercise when perceptual responses are very high, a so-called “sensory tolerance limit” may be reached after which the central motor outputs are reduced (12, 13, 42) to avoid excessive peripheral muscle exertion.

Skeletal muscle group III–IV afferents are involved in the regulation of perceptions of effort (43) and discomfort (44), which influence tolerance and motivation to exercise, although there is some debate about the finer details after a recent meta-analysis questioned their role in the perception of effort per se (45). Gandevia (4) described a sensory tolerance limit as the “threshold” beyond which the exercise may no longer be tolerated. This includes signals from exercising muscles when sensory afferents are highly stimulated, causing a negative feedback loop ending with the cessation of exercise (46). In past studies, elevated RPE was associated with early attainment of the sensory tolerance limit (13, 29), which is in line with our findings. Our results support the concept of the sensory tolerance limit by adding the possibility that afferent signals originating from muscles not directly involved in the exercise also contribute to the overall perceptions during exercise, causing exercise to terminate before the E-C coupling and cross-bridge function become impaired.

In the present study, passive stretching of a nonexercising limb consistently reduced TTF of the contralateral homologous exercising limb, but the mechanisms linking central mechanisms of fatigue were only evident when stretching was applied over DOMS. This mechanism could be explained by the combination of stretching and DOMS, thus activating both mechanoreceptors and nociceptors leading to reduced central motor drive (47). Indeed, a previous study found that intermittent static stretching of the resting knee extensors to reach up to 90% of maximal discomfort was able to elicit a reduction in MVC and VA of the contralateral leg (48), demonstrating the effects of activating both nociceptors and mechanoreceptors. In our study, a continuous stretch was applied to the resting muscles, as opposed to the intermittent protocol used by others (49). Continuous stretching can lead to a stretch tolerance effect (49) in which the sensation of stretching is reduced over time despite no change in the application of stretching, linked to a reduced nociceptive nerve endings sensitivity (50) and decreased inhibition from muscle spindles (51, 52). In this case, the presence of DOMS and pain in the early stages of the stretch may be eased by the end stages. Therefore, it is possible that different types of receptors could have been progressively active during our continuous static stretching protocol, resulting in the reduced VA.

We found no differences in performance fatigability, MVC, VA, or Q_tw,pot_ of the contralateral exercising limb when the resting limb experienced DOMS (but was not stretched) compared with CTRL. Several previous studies found a decrease in neuromuscular function after EIMD on ipsilateral muscles (34). Recent reports suggest neuromuscular impairments (53) and reduced exercise performance (28) in the contralateral homologous muscles following EIMD, demonstrating that neural pathways can provide a crossover effect for homologous muscles not directly involved in the exercise (28, 53). However, our methodology was different from those previous studies, where they provided an exercise stimulus for the muscles damaged by prior EIMD; in our study, the muscles affected by DOMS remained rested, whereas the unaffected contralateral limb exercised, and this likely accounts for the conflicting findings. For these reasons, future studies are needed to confirm the presence and the possible mechanisms of the crossover effect on the contralateral homologous limb following EIMD without any mechanical activation of the mechanonociceptors fibers.

### Perspectives and Significance

Our findings indicate that the activation of mechanoreceptors by applying a static stretch to the rested knee extensors of one leg can lead to a reduced TTF of the knee extensors of the contralateral homologous limb. When the stretch was applied to muscles also experiencing DOMS, the evidence suggests heightened central mechanisms of fatigue and further TTF reduction. This study provides an integrated physiological perspective by exploring how mechanical and nociceptive receptor activation levels affect muscle performance and neuromuscular function. From an evolutionary standpoint, the body’s ability to modulate muscle performance and fatigue through sensory feedback mechanisms is crucial for optimizing response to exercise and avoiding overexertion (5). The crossover effect observed in this study highlights the complex neural pathways involved in muscle fatigue and performance, especially when afferent activity is heightened during exercise. The sensitized mechanoreceptors and nociceptors in the stretched muscle likely contribute to the heightened perception of effort and accelerated central fatigue, as evidenced by the increased RPE during the TTF (54). This suggests that altered sensory feedback from the stretched muscle increases the perceived difficulty of the exercise, leading to premature task cessation, which is similar to findings in chronic fatigue syndrome and fibromylagia (39). The implications of this study are broad, suggesting potential applications in understanding and managing conditions where peripheral sensitization reduces exercise tolerance. For instance, strategies that manipulate sensory feedback could be developed to enhance physical performance or manage chronic pain conditions (54). In addition, understanding the central mechanisms of fatigue could inform treatments for conditions characterized by chronic fatigue and reduced exercise tolerance. Future directions for this research might focus on how afferent sensitization following EIMD affects exercise performance at both peripheral and central levels. Investigating these mechanisms could reveal how they affect exercise training in sports and exacerbate symptoms of fatigue and pain in clinical conditions. This will help to identify approaches that maximize training adaptation (i.e., individualized training), particularly in clinical settings, to accommodate individual variability and avoid exacerbating symptoms.

### Conclusions

Our findings indicate that the activation of mechanoreceptors by applying a static stretch to the rested knee extensors of one leg can lead to a reduced TTF of the knee extensors of the contralateral limb. When stretch was applied to rested muscles also experiencing DOMS 2 days after EIMD, the evidence points toward heightened central mechanisms of fatigue.

### Limitations

Our study focused on the role of mechanonociceptors, but there is a possibility that metaboreceptors were also activated by the stretch. Previous research on continuous static stretching has shown that prolonged stretching (>5 min) can reduce microvascular oxygen levels, thereby affecting muscle metabolic environment and possibly triggering metaboreceptors (55). However, the latter seems to occur when pH levels drop below 7.2 and lactate levels exceed >5 mmol (6). This is typical of moderate-intensity exercise (6) or full ischemic conditions (lasting >5 min) (56). Moreover, full ischemia does not affect muscle performance of the contralateral exercising limb (57). Therefore, the likelihood that our moderate-intensity stretching protocol achieved the necessary metabolic perturbation levels required to trigger metabo- or metabonociceptors is low. Furthermore, although the muscle metabolic milieu was perturbed following EIMD, previous research showed no changes in pH at rest and during low- to moderate-intensity exercise (58). Consequently, we may assume that activation of muscle nerve afferent during our protocol was predominantly due to mechano- and nociceptors, with minimum or negligible metaboreceptors involvement. Furthermore, we found that two of our participants reported reduced force during the stretching protocol, from ST to DOMS+ST conditions (−6 and −14 N, respectively). This might raise the possibility of a tonic stretch reflex in some people (increased force at ankle), or voluntary activation of hamstrings muscle (perhaps to lessen pressure of the force transducer against the skin at the ankle). Although the participants were familiarized and instructed to not contract the stretched leg, we cannot completely rule out partial activation of the hamstrings muscle.

## DATA AVAILABILITY

Data will be made available upon reasonable request.

## GRANTS

This project has received funding from the European Union’s Horizon 2020 research and innovation program under the Marie Skłodowska-Curie grant agreement No 801604 for F.Z. and P.D.-O. This project was also partially supported by the Brain Research Foundation Verona Onlus and by the Italian Ministry of Research and University (MIUR) 5-yr special funding to strengthen and enhance excellence in research and teaching (https://www.miur.gov.it/dipartimenti-di-eccellenza).

## DISCLOSURES

No conflicts of interest, financial or otherwise, are declared by the authors.

## AUTHOR CONTRIBUTIONS

F.Z., F.G.L., T.F., G.G., F.S., J.S.M. and M.V. conceived and designed research; F.Z., T.F. and M.V. performed experiments; F.Z., F.G.L. and P.D.-O. analyzed data; F.Z., F.G.L., G.G., M.M.O., F.S., J.S.M. and M.V. interpreted results of experiments; F.Z. prepared figures; F.Z., F.G.L., J.S.M., and M.V. drafted manuscript; F.Z., F.G.L., T.F., G.G., M.M.O., P.D.-O., J.S.M., and M.V. edited and revised manuscript; F.Z., F.G.L., T.F., G.G., M.M.O., F.S., P.D.-O., J.S.M., and M.V. approved final version of manuscript.
